# Advances in Nanodelivery of Green Tea Catechins to Enhance the Anticancer Activity

**DOI:** 10.3390/molecules26113301

**Published:** 2021-05-31

**Authors:** Yike Jiang, Ziyi Jiang, Lan Ma, Qingrong Huang

**Affiliations:** 1 Shenzhen Bay Laboratory, Institute of Biomedical Health Technology and Engineering, Shenzhen 518132, China; malan@sz.tsinghua.edu.cn; 2 Institute of Biopharmaceutical and Health Engineering, Tsinghua Shenzhen International Graduate School, Tsinghua University, Shenzhen 518055, China; jiang-zy20@mails.tsinghua.edu.cn; 3 State Key Laboratory of Chemical Oncogenomics, Tsinghua Shenzhen International Graduate School, Tsinghua University, Shenzhen 518055, China; 4 Department of Food Science, Rutgers University, 65 Dudley Road, New Brunswick, NJ 08901, USA

**Keywords:** green tea, phenolic compounds, catechins, anticancer activity, nanodelivery system

## Abstract

Cancer is one of the leading causes of death globally. A variety of phenolic compounds display preventative and therapeutic effects against cancers. Green teas are rich in phenolics. Catechins are the most dominant phenolic component in green teas. Studies have shown that catechins have anticancer activity in various cancer models. The anticancer activity of catechins, however, may be compromised due to their low oral bioavailability. Nanodelivery emerges as a promising way to improve the oral bioavailability and anticancer activity of catechins. Research in this area has been actively conducted in recent decades. This review provides the molecular mechanisms of the anticancer effects of catechins, the factors that limit the oral bioavailability of catechins, and the latest advances of delivering catechins using nanodelivery systems through different routes to enhance their anticancer activity.

## 1. Introduction

Drinking tea (Camellia sinensis) has a long history that can be traced back to over 4700 years ago in ancient China [1]. Teas have become the second most widely consumed beverage worldwide nowadays. Depending on the degree of enzymatic fermentation, teas are generally classified into green teas (non-fermented), oolong teas (semi-fermented), and black teas (fermented). They account for about 20%, 2%, and 78% of total tea consumption, respectively [2].

Drinking green tea is popular in east Asian countries. As a traditional medicinal herb in China, the medicinal effects of green teas have been recorded in ancient Chinese medical books Shen Nong’s Herbal Classic and Compendium of Materia Medica, etc. Modern research has revealed that drinking green tea is beneficial for health. Epidemiological studies demonstrate that regular consumption of green tea is associated with reduced risks of death from cardiovascular diseases and certain types of cancers [3,4]. The beneficial health effects of green tea mainly stem from the phenolic components. Great efforts have been made in recent decades to clarify the underlying mechanisms of these bioactivities [5,6,7,8]. Thousands of articles are published every year in related areas. However, some in vitro bioefficacies of green tea phenolics are compromised or even ineffective in the human body [4,8], which is highly related to their poor oral bioavailability.

Chemists, pharmacologists, biologists, and food scientists are using various approaches to enhance the bioavailability of green tea phenolics, such as structural modification, using intestinal permeability enhancers, and the modulation of metabolic activities [9,10]. Nanodelivery systems emerge as powerful tools that can enhance the stability, solubility, bioavailability, and bioefficacy of the payload. They can also achieve targeted delivery when modified with targeting molecules. We herein review the anticancer activity and corresponding molecular basis of the most dominant green tea phenolics, the catechins, and analyze the factors that reduce their oral bioavailability and highlight the recent advances in improving the anticancer activity of green tea catechins by using rationally designed nanodelivery systems through different routes.

## 2. Phenolics in Green Teas

Tea leaves are rich in phenolics. Flavan-3-ols are the dominant phenolics in teas, constituting about 25% of the dry weight of a tea leaf. Flavonols and their glycosides, phenolic acids, and other polyphenols also exist in the tea leaf as the minor phenolic component [11,12].

**Figure 1:**
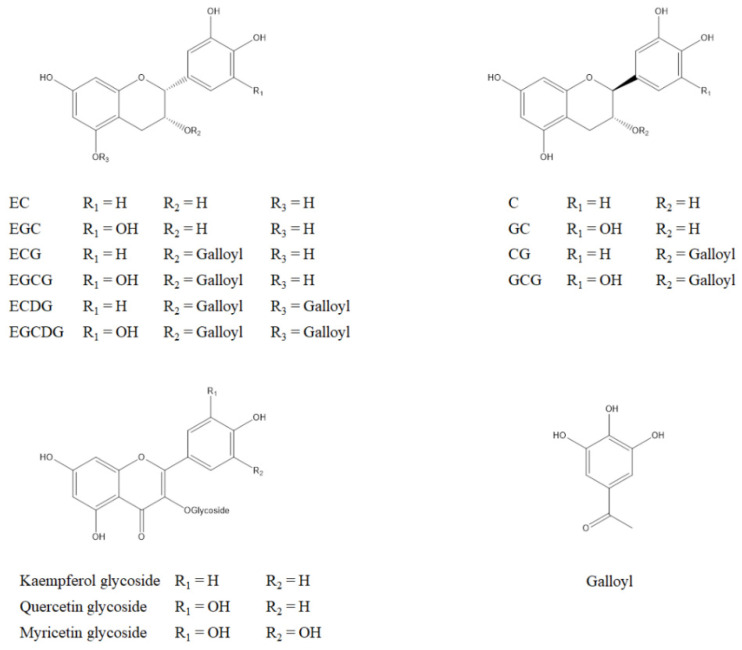
Structures of major phenolics exist in green tea: EC: (−)-epicatechin; EGC: (−)-epigallocatechin; ECG: (−)-epicatechin gallate; EGCG: (−)-epigallocatechin gallate; ECDG: epicatechin digallates; EGCDG: epigallocatechin digallates; C: (+)-catechin; GC: gallocatechin; CG: catechin gallate; GCG: gallocatechin gallate.

As green teas are processed by steaming or pan-frying immediately after harvesting to inactivate enzymatic fermentation, flavan-3-ols in green teas mainly exist in the monomeric forms, which are generally referred to as catechins. Unlike most of the other phenolics, catechins occur naturally without the attachment of sugar moieties. (−)-Epigallocatechin gallate (EGCG) is the most dominant catechin, constituting 50–80% of total catechins [2]. Other major catechins include (−)-epicatechin (EC), (−)-epigallocatechin (EGC), and (−)-epicatechin gallate (ECG). These four catechins have two chiral centers at C-2 and C-3 positions, but they all occur in *2R*, *3R* (2,3-*cis*) forms. They are differentiated by the substitution of a hydroxyl group at the C-5′ position on the B-ring and a galloyl group at the 3-*O* position on the C-ring (Figure 1). Other catechins including gallocatechin gallate (GCG), gallocatechin (GC), catechin gallate (CG), (+)-catechin (C), epigallocatechin digallates (EGCDG), and epicatechin digallates (ECDG) (Figure 1) and their derivatives exist in small quantities in green teas [2,12]. These catechins have been discovered long before. EC, ECG, and EGC were discovered in the early 20th century [13,14,15], and EGCG was first reported by Bradfield et al. in 1948 [16]. These four major catechins have been extensively investigated for decades. ECDG and EGCDG were discovered later by Coxon et al. in 1972 [17] and they are relatively less studied compared to EC, ECG, EGC, and EGCG.

Also, flavonols including kaempferol, quercetin, myricetin, and their glycosides (Figure 1) are present in green teas as minor but still important phenolic components [12].

## 3. Anticancer Activity of Green Tea Catechins

**Figure 2:**
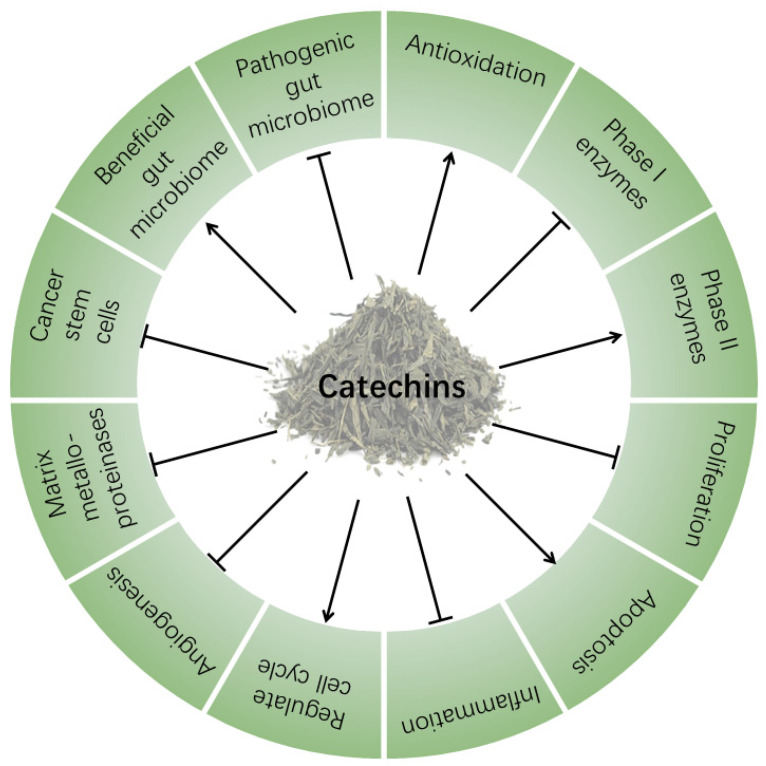
Anticancer mechanisms of catechins. The arrows and T-shaped lines signify positive and negative regulations, respectively.

Cancers are the most life-threatening diseases currently. Breast, lung, prostate, skin, colon, stomach, and liver are the sites where cancers most frequently occur [18]. Green tea catechins have shown therapeutic effects against multiple types of cancers in vitro and in vivo [5,19]. Herein, we review the major cancer-inhibition mechanisms of catechins (Figure 2).

### 3.1. Antioxidation

Redox homeostasis is important for a normal physiological steady state by maintaining normal cell metabolism, survival, proliferation and differentiation, angiogenesis, immune defense, etc. [20,21]. The human body has a defense system to maintain redox homeostasis by regulating reactive oxygen species (ROS) with enzymatic antioxidants (e.g., superoxide dismutase, glutathione peroxidase, and catalase), and non-enzymatic antioxidants (e.g., vitamin C, vitamin E, glutathione, carotenoids, and phenolics) [22]. Over-generation of ROS perturbs the balance and causes deleterious oxidative stress, leading to damage to DNA, proteins, and lipids, which is associated with cancer development [23,24]. The phenolic compounds, as represented by catechins, are potent natural antioxidants. Antioxidation is an important anticancer mechanism of catechins. They can diminish excess ROS by various mechanisms including scavenging free radicals, chelating metal ions, regenerating endogenous antioxidants, and regulating free radical-generating enzymes and antioxidant enzymes [25]. Also, several carcinogenic pathways such as MAPK, nuclear factor-κB (NF-κB), and PI3K pathways are activated in response to oxidative stress [24]. Catechins can inhibit cancer development by regulating these oxidative stress-related pathways [26]. Wolfe et al. investigated the relationship between the structure and the cellular antioxidant activity of four major catechins. The presence of the galloyl group remarkably increased the cellular antioxidant activity of EGCG and ECG. The extra hydroxyl group in the B-ring also provided higher antioxidant activities to EGCG and EGC. As a result, they reported that EGCG had the most potent cellular antioxidant activity followed by ECG, EGC, and EC [27].

### 3.2. Regulation of Drug Metabolizing Enzymes

The drug metabolizing enzymes including phase I and phase II enzymes play important roles in cancer development. The phase I enzymes are involved in the phase I reactions such as oxidation, reduction, and hydrolysis. Phase II enzymes participate in the phase II conjugation reactions such as glucuronidation, sulfation, methylation, acetylation, glutathione conjugation, or amino acid conjugation [28]. Catechins can regulate phase I and phase II enzymes to inhibit carcinogen-induced DNA damages. Phase I metabolizing enzymes catalyze the addition of polar groups to carcinogens, which subsequently react with DNA. Inhibition of phase I metabolizing enzymes, particularly cytochrome P450 (CYP450) is an important strategy to block carcinogenesis in the initiation stage of cancer development. It was reported that EGCG and ECG can inhibit CYP450 1A1 (CYP1A1), CYP1A2, CYP3A4, CYP2A6, CYP2C19, and CYP2E1 [6,29,30]. Phase II conjugating enzymes play key roles in detoxifying the xenobiotics by conjugating the polar groups that are introduced by the phase I enzymes and thereby prevent damages of the genome and initiation of carcinogenesis. It was reported that catechins effectively up-regulated the activities of antioxidant and phase II enzymes in different organs of mice [31]. Antioxidant response element (ARE) is a specific DNA promoter-binding region that presents in the 5′-flanking regions of genes encoding antioxidant enzymes and phase II enzymes. Nuclear factor-erythroid 2p45 (NF-E2)-related factor 2 (Nrf2) is a key transcription factor that binds to ARE under stress (such as oxidative stress and xenobiotic chemicals-induced stress) to induce the transcription of phase II enzymes [32,33]. It was reported that EGCG and ECG can stimulate Nrf2 [34,35]. Kelch-like ECH-associated protein 1 (Keap1) is a cytoplasmic protein that binds to Nrf2 and weakens the transcriptional activity of Nrf2. EGCG may facilitate the dissociation of Nrf2 with Keap1 [36].

### 3.3. Inhibition of Proliferation

Uncontrolled proliferation is a hallmark of carcinogenesis. Several signaling pathways are involved in cancer cell proliferation. The MAPK, PI3K, and NF-κB pathways, and activator protein-1 (AP-1) are important targets for catechins to inhibit proliferation. Dysregulation of the MAPK and PI3K pathways leads to uncontrolled proliferation. NF-κB is a transcription factor that is involved in regulating the expression of cytokines, inducible NO synthase, cyclooxygenase-2 (COX-2), growth factor, and inhibitors of apoptosis [12]. Aberrant activation of NF-κB is correlated with enhancing proliferation and suppressing apoptosis [33]. AP-1 is another important transcription factor that is implicated in the regulation of the expression of genes that are involved in proliferation and apoptosis. Activation of AP-1 is associated with tumor promotion. It was reported that green tea polyphenols and EGCG inhibited the proliferation and induced apoptosis of breast cancer cells both in vitro and in vivo [37]. Also, EGCG effectively inhibited the activities of NF- κB, AP-1, as well as the PI3K and MAPK pathways in multiple cancer cell lines [33,38,39].

### 3.4. Induction of Apoptosis

Under normal conditions, apoptosis maintains the homeostasis of tissue development, while during carcinogenesis the normal apoptosis is suppressed. Aberrant apoptosis of cancer cells stems from overexpression of growth-promoting oncogenes and anti-apoptotic proteins, such as Ras and Bcl-2, or arrestment of pro-apoptotic proteins, such as p53 and Bax [6]. Therefore, inducing apoptosis of the damaged cells is potentially useful to halt tumorigenesis. EGCG-induced apoptosis was reported in various cancer cell models [40]. EGCG also increased the expression of p53 and Bax and lowered the levels of Bcl-2 and c-Myc in vivo [41]. ECG was able to enhance the expression of p53 and at the same time suppress the expression of Bcl-2 in the human lung cancer cell line [6].

### 3.5. Antiinflammation

Chronic inflammation is closely associated with cancer development [42]. Research has found that pro-inflammatory factors such as NF-κB, tumor necrosis factor (TNF), interleukins (IL), and COX-2 are involved in tumorigenesis. They also participate in the creation of the inflammatory microenvironment of tumors [42,43]. ROS and reactive nitrogen species (RNS) are produced in inflammation. Therefore, the antioxidant ability of catechins is one of the anti-inflammatory mechanisms in this sense. NF-κB is associated with activating the expression of cytokines, chemokines, enzymes, and adhesion molecules. In normal conditions, the activity of NF-κB is blocked by its inhibitory protein IκB, while under inflammatory states, IκB is phosphorylated by IκB kinase, which leads to the release of NF-κB and promotes the transcription of pro-inflammatory genes. Flavan-3-ols, including catechin, epicatechin, and dimeric procyanidin can inhibit NF-κB activation [44]. Overexpression of COX, especially COX-2, can catalyze the conversion of arachidonic acid to prostaglandins, which promotes inflammation. COX-2 is downregulated in multiple types of cancers by catechins [19]. The lipoxygenases (LOX), particularly 5-LOX and 12-LOX play important roles in inflammatory diseases [45]. Research indicated that EC and EGCG can inhibit the activities of 5-LOX, which could contribute to the anti-inflammatory and anticancer effects of catechins [46].

### 3.6. Regulation of Cell Cycle

Cyclins-dependent kinases (CDKs) are the key molecules that regulate the cell cycle. EGCG can downregulate the expression of cyclin D1, CDK 4, and CDK 6, and upregulate cyclin kinase inhibitors p21, p27, p16, and p18 in human epidermoid carcinoma cells, resulting in the arrest of G0/G1 phase cell cycle [47]. Also, EGCG arrested the G1 phase of the breast cancer cell cycle by lowering the expression of cyclin D, cyclin E, CDK 4, CDK 1, and proliferating cell nuclear antigen (PCNA). Similarly, EGCG arrested the G1 phase of the intestinal epithelial cell cycle by suppressing cyclin D1 expression [48]. EGCG also decreased the level of cyclin E and the activity of CDK 2 in cervical cells [49].

### 3.7. Inhibition of Cancer Metastasis

Metastasis is implicated in 90% of cancer-related deaths [50]. It is still one of the major reasons for the poor prognosis of cancers. Angiogenesis is a critical step for solid tumor growth and metastasis. Thus, inhibition of angiogenesis is a strategy to suppress tumor growth and metastasis. Vascular endothelial growth factor (VEGF) is an important inducer of angiogenesis [51]. EGCG not only downregulates the expression of VEGF but also inhibits the binding of VEGF to its receptor [52,53,54], which contribute to the anti-angiogenic and anti-metastatic effects. Matrix metalloproteinases (MMPs) play important roles in tumor cell invasion and metastasis [55]. There are 23 types of MMPs in the human body that are generally grouped into collagenases, gelatinases, stromelysins, matrilysins, and membrane-type MMPs based on the substrates. Overexpression of MMPs, particularly MMP-1, MMP-2, MMP-3, MMP-7, MMP-9, and MMP-12 are commonly reported in cancers [56]. Suppressing the expression of MMPs is an important mechanism of inhibiting metastasis for phenolics. It was reported that EGCG inhibited metastasis of human pancreatic cancer cells by lowering the levels of MMP-2, MMP-7, MMP-9, and MMP-12 [52]. Also, ECG downregulates MMP-2, MMP-9, and MMP-7 activities in diverse cells [6]. Epithelial-mesenchymal transition (EMT) also contributes to tumor metastasis [57]. EGCG can inhibit EMT in lung cancer cells through deacetylation of Smad2 and Smad3 [58] and downregulation of phosphorylated Smad2 and Erk1/2 [59]. In pancreatic cancer cells, EGCG can inhibit EMT via the inhibition of the Akt pathway [60].

### 3.8. Inhibition of Cancer Stem Cells

Cancer stem cells (CSCs) are a subgroup of cancer cells that have the abilities of self-renew and differentiation [61]. Accumulating evidence revealed that CSCs contribute greatly to the development, recurrence, metastasis, chemo- and radiotherapy resistance of cancers [62,63,64]. CSCs have become a new target of cancer therapy nowadays. The Hedgehog, Notch, Wnt, MAPK, PI3K, TGF-β, and JAK-STAT pathways play important roles in maintaining CSCs [65]. It was reported that EGCG can inhibit the CSCs through suppression of the Wnt/β-catenin [66], Notch [67], and Hedgehog [68] pathways, and therefore, inhibit the CSCs.

### 3.9. Regulation of Gut Microbiota

The relationship between gut microbiota and cancer development has been actively investigated in recent decades [69,70]. Gut microbiota participates in cancer development by affecting metabolism, inflammation, and adaptive immunity [71]. It is clear now that diet can influence the composition and metabolism of gut microbiota [72]. Scientists realized that the anticancer efficacy of catechins also comes from their interactions with human gut microbiota. On the one hand, human gut microbiota catalyzes the biotransformation of catechins. Catechins that are not absorbed in the small intestine go to the gut and are metabolized by gut microbiota. A wide variety of catechin metabolites catalyzed by gut microbiota have been identified so far in vitro and in vivo [73]. The metabolites display anticancer effects in different cancer cell models with various mechanisms [73]. For example, 3,4-dihydroxyphenylpropionic acid, the metabolite of C/EC/EGC/ECG/GC/CG, significantly inhibited the secretion of proinflammatory cytokines TNF-α, IL-6, and IL-1β [74]. On the other hand, catechins and their metabolites regulate the composition of gut microbiota and consequently achieve anticancer effects. Green tea polyphenols promote the growth of beneficial microbes (e.g., *Bifidobacterium spp.*, *Lactobacillus spp.*) and inhibit the growth of pathogenic microbes (e.g., *Clostridium perfringens*, *Clostridium difficile*, *Bacteroides spp.*, *E. coli O157:H7*, *H. pylori*) [8,72]. *H. pylori* infection is associated with gastric cancer [75]. Intake of green tea, in this sense, would be able to inhibit gastric cancer through the inhibition of *H. pylori*.

Some of the in vitro and in vivo inhibitory effects of catechins against lung, breast, ovarian, gastric, colon, pancreatic, liver, bladder, and prostate cancers are listed in Table 1. Although the anticancer effects of catechins have been extensively investigated, the structure–anticancer activity relationship of catechins is inconclusive so far. It is generally recognized that the anticancer activity of catechins is EGCG > ECG > EGC > EC [76,77], however, ECG sometimes displays an equal or more potent anticancer activity than EGCG [6]. Thus, more studies comparing the inhibitory activity of catechins against different types of cancers are required to conclude the structure–anticancer activity relationship of catechins. Also, the cytotoxicity of catechins towards normal cells should also be considered as a high dosage of catechins could be toxic to cancer cells, and at the same time toxic to normal cells and tissues. In a series of toxicological studies, Isbrucker et al. reported that a single oral consumption of up to 2000 mg/kg of EGCG (91.9% purity) or oral administration of up to 1200 mg/kg of EGCG (80% purity) for 10 days did not induce micronuclei formation in bone marrow cells of mice. A higher plasma concentration of EGCG in rats did not induce genotoxicity [78]. However, an oral dose delivering 2000 mg/kg of EGCG (90% purity) resulted in the death of rats [79]. Long-term consumption of up to 500 mg/kg/day of EGCG (77% purity) for 13 weeks in rats did not show toxicity [79]. Lambert et al. pointed out that high doses of EGCG could be toxic to the liver, kidneys, and intestine [80]. Hence, the adverse effects induced by high doses of catechins should not be neglected when commending their anticancer effects. 

## 4. Oral Bioavailability of Catechins

Oral consumption is the natural way of ingesting green tea catechins. Oral bioavailability is important for the orally consumed biofunctional food ingredients (nutraceuticals). Bioavailability, defined by the Food and Drug Administration, is the rate and extent to which a therapeutic agent is absorbed and available to the site of action [10]. Thus, oral bioavailability is crucial for the therapeutic dosage of catechins at tumor sites and determines their anticancer activities. As we have previously reviewed, the oral bioavailability of biofunctional food ingredients (nutraceuticals) is regulated by the bioaccessibility to intestinal cells, intestinal permeability, and organ metabolism [10]. Catechins have to be released into the small intestinal lumen, solubilized or dispersed in the intestinal fluid to be accessible for the intestinal epithelial cells, then be absorbed through the small intestine followed by a series of hepatic metabolisms before entering systematic circulation [10].

Catechins are generally water-soluble, thus, they are bioaccessible to the intestinal cells.

The intestinal permeability can be described by Lipinski’s rule of five, which predicts poor absorption or permeability when half or more of the following four criteria are met: (1) molecular weight (M.W.) > 500 g/mol, (2) log P > 5 or Mlog P > 4.15, (3) has more than five hydrogen bond donors (sum of OHs and NHs), (4) has more than 10 hydrogen bond acceptors (sum of Ns and Os) [105].

Among four major catechins, EGC and ECG have more than five hydrogen bond donors. EGCG has two unfavorable parameters, i.e., 8 hydrogen bond donors and 11 hydrogen bond acceptors (Table 2). These hydrogen donors and acceptors can associate with water molecules to form large hydration shells around catechins molecules, making catechins less likely to penetrate through intestinal epithelial cells [106]. The intestinal permeability of EGCG assessed by the Caco-2 monolayer transport experiment show extremely low apparent permeability coefficients (P_app_), in 10^−7^ to 10^−8^ cm/s order of magnitudes (Table 2).

Moreover, green tea phenolics are generally unstable and undergo rapid degradation in the alkaline intestinal environment. More than 75% loss of the catechins mixtures was observed in a slightly alkaline solution (pH 7.4) within the first half an hour [108]. Individual catechins are not equally resistant to alkaline-induced degradation, with the order of stability as follows: EC > ECG > EGCG > EGC. The tri-hydroxyl groups on the B-ring might be more susceptible to pH-induced degradation than the catechol structure (3′,4′-dihydroxyl benzene) of the B-ring [108,109]. Auto-oxidation is an important degradation pathway of EGCG in alkaline conditions, particularly at low EGCG concentrations. The oxidation products of EGCG in the cell culture medium are theasinensin A, P2, and other dimers, which are also unstable and undergo further transformations in the alkaline solution [110]. The degradation products of catechins in the alkaline intestinal environment usually have higher M.W. and more hydrogen bond donors and acceptors, leading to even lower intestinal permeability.

On the other hand, green tea catechins and metabolites are the substrates of ATP-binding cassette transporters, including multidrug resistance-associated proteins and P-glycoprotein, which efflux catechins back into the intestinal lumen [107,111]. The efflux of catechins characterized by the Caco-2 monolayer experiment demonstrates that the non-gallate catechins (EC and EGC) have higher degrees of efflux (Table 2). Thus, the significant efflux further decreases the intestinal permeation of green tea phenolics.

Another factor that limits the oral bioavailability of catechins is their specific organ metabolisms. Catechins undergo intestinal and hepatic metabolisms. Methylation, sulfation, and glucuronidation catalyzed by the catechol-*O*-methyltransferase (COMT), sulfotransferases, and UDP-glucuronosyltransferases, respectively, are the dominant hepatic metabolic pathways for catechins [2]. EC mainly undergoes sulfation (66%) and glucuronidation (33%). EGC is mainly metabolized by glucuronidation (57–71%) and sulfation (23–36%) [106]. EGC-3′-*O*-glucuronide is the major glucuronidated product of EGC. EGC is also methylated into 4′-*O*-methyl EGC [112]. EGCG can be converted into 4″-*O*-methyl EGCG and 4′, 4″-di-*O*-methyl-EGCG by liver COMT [2]. With the advances in detection techniques, new hepatic metabolic pathways may be revealed. These biotransformations significantly decreased the amount of parent catechins entering systemic circulation, thus, further reduces their bioavailabilities.

**Figure 3:**
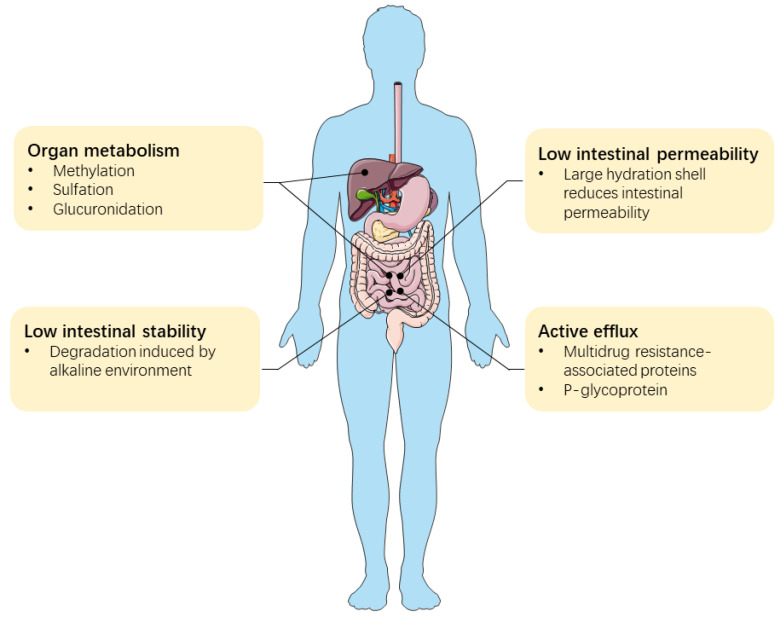
Unfavorable features contribute to the low oral bioavailability of catechins.

These unfavorable features collectively lead to the poor oral bioavailabilities of green tea catechins (Figure 3). It was reported that only 0.16%, 0.58%, and 1.1% of total EGCG, EGC, and EC from orally administered green tea (20 mg green tea solids/kg bodyweight) were detected in human plasma at the time point (T_max_) when the maximum plasma concentration (C_max_) was reached. Although EGCG is the most dominant type of catechin in green tea, its C_max_ was lower than EGC and EC. The area under the curve (AUC) of EGCG was close to EC, but still much lower than EGC [113]. The mean concentration of EGCG in the plasma in this study was only 0.17 μM, much lower than the effective therapeutic concentrations of EGCG in vitro [113]. Studies indicate that green tea consumption is weakly associated with the risks of certain types of cancers, such as gastric cancer [114], breast cancer [115], and liver cancer [116]. The unmet therapeutic dosages of catechins that stem from low oral bioavailability contribute greatly to the compromised anticancer effects in these types of human tumors.

## 5. Nanodelivery of Green Tea Catechins

In recent decades, interdisciplinary efforts have been devoted to solving the intrinsic defects and maximizing the benefits that are associated with green tea catechins. Delivery of catechins with nanocarriers emerged as a promising strategy to enhance the stability, bioavailability, and bioefficacies (e.g., anticancer, anti-inflammation, and antioxidation effects) of catechins. Thus, it becomes an active research area in the pharmaceutical, food science, and nutraceutical fields.

### 5.1. Nanocarriers for Encapsulation and Delivery of Green Tea Catechins

With the development of material sciences, carriers composed of different materials have been developed to encapsulate and deliver catechins. The size of these carriers varies from nanometers to micrometers. The carriers in the nanometer range are of particular importance because of their unique properties such as superior tumor accumulation ability. Polymer-based and lipid-based nanoparticles (NPs) are the most commonly used encapsulation and delivery systems for catechins.

#### 5.1.1. Polymer-Based NPs

The polymer-based nanodelivery systems can be generally grouped into natural polymeric NPs and synthetic polymeric NPs.

Proteins are the most commonly used natural polymers for generating nanodelivery systems for catechins. Proteins interact with catechin through non-covalent bonds including hydrogen bonding, hydrophobic interactions, and van der Waals interactions [117], and subsequently encapsulate catechins. Wu et al. developed the EGCG-loaded β-lactoglobulin NPs, which enhanced the inhibitory effect of EGCG against multiple types of cancer cells, particularly the human melanoma A375 cells and human esophageal carcinoma TE-1 cells. The NPs enhanced the anticancer effect of EGCG through increased apoptosis, suppressed proliferation, and appreciable cell cycle arrest [118]. Other proteins such as hordein and gelatin (Table 3) were also used to encapsulate catechins, which displayed different types of enhancements that may potentially contribute to an improved anticancer effect.

Polysaccharides are another group of natural polymers for encapsulation and nanodelivery of catechins. Chitosan (CS) is the most commonly used polysaccharide for preparing the encapsulation and delivery systems for catechins. In a study, Zeng et al. loaded EGCG in folate-modified CS NPs and they reported that these CS NPs significantly enhanced the inhibitory effect against human breast cancer MCF-7 cells through upregulation of PTEN, p21, and Bax, and downregulation of p-PDK1, p-AKT, Cyclin D1, and Bcl-2 [125]. Other polysaccharides such as alginate [143], gum Arabic [144], and cyclodextrin [145] have also been used to encapsulate catechins.

Proteins and polysaccharides are often used simultaneously in a single system for the encapsulation of catechins (Table 3). At a specific pH range where proteins and polysaccharides are oppositely charged, they are strongly associated primarily through electrostatic interactions, forming the complex coacervates. Catechins are encapsulated in the complex coacervates during the complexation process. Dai et al. recently encapsulated EGCG in the CS/β-lactoglobulin nanocomplexes. The CS/β-lactoglobulin nanocomplexes enhanced the cellular antioxidant activity of EGCG in vitro [126]. Kumar et al. proved that oral delivery of green tea polyphenol-loaded bovine serum albumin (BSA)/CS complexes significantly improved the radioprotective effects of green tea polyphenols in vivo by restoring the redox status through the Nrf2-ERK pathway and reducing Bax expression [130]. The complex coacervates composed of proteins and polysaccharides have the advantages of both components. However, complex coacervates are sensitive to pH change. When they are used as oral delivery systems, the sharp pH change upon passing through the gastrointestinal (GI) tract is a great challenge as the pH change may cause dissociation of the complex coacervates and burst release of catechins. Using crosslinkers to chemically crosslink the complex coacervates is a strategy to increase the pH stability of complex coacervates. Hu et al. used genipin to crosslink the EGCG-loaded CS/caseinophosphopeptide nanocomplexes. The crosslinking strategy slowed the burst release of EGCG from nanocomplexes in the simulated gastric environment [146]. Proteins and polysaccharides are sometimes conjugated as co-polymers and they self-assemble into NPs to encapsulate catechins. Li et al. conjugated ovalbumin with dextran via the Maillard reaction. EGCG was loaded in the ovalbumin-dextran NPs, which were further crosslinked by glutaraldehyde. These NPs significantly enhanced the in vitro intestinal permeability of EGCG as confirmed by the Caco-2 monolayer model [129].

A variety of proteins, polysaccharides, and their complex coacervates have been investigated as nanocarriers for encapsulation of catechins and they have displayed great improvements in the stability, intestinal permeability, oral bioavailability, anticancer activity, etc., of catechins (Table 3).

Natural polymers, especially polysaccharides, usually are polydisperse in molecular weight. Polymeric NPs composed of natural polymers often show polydispersity. NPs composed of synthetic polymers, such as poly(lactic-co-glycolic acid) (PLGA) are generally more homogeneous. Also, synthetic polymers provide more opportunities for modifications, offering various functionalities. The catechin-loaded PLGA NPs have shown effective improvements in the in vivo anticancer effect of catechins (Table 4). Zhang et al. recently loaded EGCG in PLGA NPs and they reported that the EGCG-loaded PLGA NPs displayed significantly stronger inhibition against patient-derived lung cancer xenograft growth in vivo than free EGCG [147].

#### 5.1.2. Lipid-Based NPs

Another group of commonly used encapsulation systems for the nanodelivery of catechins is lipid-based NPs. Solid lipid nanoparticles (SLNs) are the most frequently seen lipid-based delivery system for catechins. It was reported that SLNs can significantly improve the oral bioavailability of EGCG in rats. Also, compared to oral administration of free EGCG, the EGCG-loaded SLNs significantly increased the tissue levels of EGCG (total EGCG) in the brain, kidney, liver, and spleen. The spleen contained the highest concentration of EGCG after oral consumption of the EGCG-loaded SLNs while the kidney had the highest level of EGCG from oral consumption of free EGCG. Acute and sub-chronic toxicities were not observed after oral administration of the EGCG-loaded SLNs [153].

### 5.2. Encapsulation Methods

Catechins are usually loaded in nanocarriers through physical encapsulation and/or chemical conjugation.

Physical encapsulation is the most frequently used strategy for the encapsulation of catechins. Catechins can interact with proteins and polysaccharides through hydrophobic interactions, hydrogen bonding, and van der Waals interactions [117,154,155]. Catechins are usually entrapped in protein-, polysaccharide-, and protein/polysaccharide complex coacervate-based NPs through these non-covalent interactions. In a series of studies related to β-lactoglobulin-based NPs, EGCG solution was added into preheated β-lactoglobulin solution followed by cooling to room temperature [118,119,156,157]. Heat treatment in these studies induced the unfolding of β-lactoglobulin and exposure of the inner hydrophobic domains, which allowed strong hydrophobic interactions and hydrogen bonding between EGCG and β-lactoglobulin. Therefore, EGCG was encapsulated in the β-lactoglobulin NPs. Ionic crosslinking is often used in CS-based nanoencapsulation. Catechins are usually pre-mixed with CS to form complexes through non-covalent interactions. These cationic complexes then ionically interact with anionic molecules such as TPP to physically entrap catechins in the CS/TPP NPs (Table 3). Similarly, the catechin-loaded protein/polysaccharide complex coacervates are usually prepared by physically mixing catechins with one type of polymer followed by the addition of the other type of polymer. Catechins are thus physically entrapped in the complex coacervates upon ionic crosslinking between proteins and polysaccharides [126,158].

PLGA NPs can be prepared by multiple strategies such as nanoprecipitation, emulsion/solvent evaporation, dialysis, and film-casting [159]. Physical entrapment of catechins in the PLGA NPs is commonly achieved by emulsion/solvent evaporation. Typically, catechin solution (water phase) is added into PLGA solution (organic phase) to form the primary water-in-oil emulsion, which was then added into another aqueous solution (typically polyvinyl alcohol solution) to form the water-in-oil-in-water emulsion. The organic phase is then removed to form the catechin-loaded PLGA NPs [147,160].

Catechin can be physically loaded in lipid-based NPs by various methods. De Pace et al. encapsulated EGCG in nanoliposomes by using the conventional thin-film hydration method [139]. Zou et al. fabricated the tea polyphenol-loaded nanoliposomes by combining an ethanol injection method with dynamic high-pressure microfluidization [138]. SLN can be prepared by different methods such as high-pressure homogenization, microemulsion breaking technique, solvent-emulsification diffusion technique, solvent injection method, etc. [161]. The double emulsion method is commonly used for SLNs loaded with hydrophilic drugs and biological molecules [161]. Encapsulation of catechins in SLNs often employs the double emulsion method [136,137]. Phase inversion [140] and hot homogenization methods [141] were also used to prepare EGCG-loaded nanostructured lipid nanocarriers.

Catechins are sometimes chemically conjugated to nanocarriers. Moreno-Vasquez et al. conjugated EGCG to CS, which self-assembled into NPs upon nanoprecipitation [162]. Mukherjee et al. conjugated green tea polyphenols or EGCG to gold NPs by a reduction method. These green tea polyphenol- or EGCG-conjugated gold NPs were highly toxic to cancer cells (Ehrlich’s Ascites Carcinoma and MCF-7 cells) but nontoxic to normal mice hepatocytes [163].

### 5.3. Delivery Strategies

The catechin-loaded NPs can be delivered to tumors via different routes, generally including non-invasive and invasive routes. Each delivery strategy has its strengths and limitations. We herein review the major delivery strategies of catechins for cancer therapy.

#### 5.3.1. Non-Invasive Delivery Strategies

The non-invasive delivery strategies are appealing because they provide painless administration of the therapeutic agents. Hence, they have high patient/customer compliance.

##### Oral Delivery

Oral delivery is convenient and non-invasive. The nano-formulated catechins designed for oral administration can be used as therapeutic drugs or fortified in foods as the functional ingredient for cancer prevention.

Improving intestinal stability and intestinal permeability are the two main strategies to maximize the oral bioavailability of catechins and consequently enhance their anticancer efficacy. As summarized in Table 3, a variety of encapsulation systems can increase the stability of catechins in neutral to slightly alkaline conditions, which resemble the physiological conditions in the small intestine.

**Figure 4:**
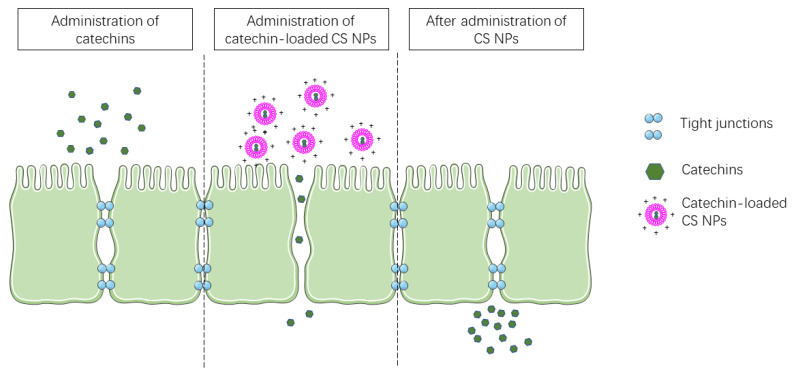
Chitosan-based nanoparticles reversibly open the tight junctions between intestinal epithelial cells.

Integrating permeation enhancers into nanodelivery systems is a feasible way to increase the intestinal permeability of catechins. CS is one of the most commonly used biomaterials for engineering nanodelivery systems. As a cationic polymer, CS prolongs the retention time in the GI tract by interacting with the negatively charged mucus layer [164,165]. Also, CS is an intestinal permeation enhancer. It was proved that CS can reversibly and transiently open the tight junctions between small intestinal epithelial cells, thus, enhance the paracellular transport [166] (Figure 4). Hu et al. showed that the CS/caseinophosphopeptide nanocomplexes can effectively enhance the in vitro intestinal permeability of EGCG [158]. Dube et al. reported that the CS/tripolyphosphate (TPP) NPs significantly enhanced the oral bioavailability of EGCG in mice. The AUC of EGCG in the CS-based NPs was 1.5-fold of the EGCG solution [167]. Khan et al. found that oral administration of the EGCG-loaded CS/TPP NPs can significantly inhibit prostate tumor development in vivo [123]. These CS-based nano-formulated EGCGs significantly decreased the expression of serum prostate-specific antigen, a widely accepted biomarker of prostate cancer. Also, poly (ADP-ribose) polymerase (PARP) cleavage, upregulation of the proapoptotic protein Bax, and downregulation of the antiapoptotic protein Bcl-2, enhanced activation of caspases-3, -8, and -9, decreased expression of the proliferation biomarkers Ki-67 and PCNA, reduced levels of the angiogenesis biomarkers CD31, and VEGF occurred in the prostate tumor upon oral administration of the nano-formulated EGCG [123]. Also, using CS/TPP NPs, Siddiqui et al. reported the enhanced anti-melanoma effect of EGCG in vivo via regulation of Bax/Bcl-2 to an apoptosis-favorable ratio, cleavage of PARP, cell cycle arrest, and inhibition of proliferation [124].

Delivering the catechins-loaded NPs through oral administration is advantageous for gastrointestinal cancers’ therapy as the orally delivered NPs pass through the GI tract.

Lin et al. developed the NPs composed of fucose-conjugated CS and PEG-conjugated CS complexes with gelatin for oral delivery of EGCG [127]. This EGCG-loaded system significantly decreased VEGF expression level in MKN45 human gastric cancer cells. Moreover, after oral administration, the EGCG-loaded NPs significantly inhibited gastric tumor growth in the tumor-bearing mice through enhanced apoptosis [127].

Targeting molecules are sometimes modified to the surface of orally delivered nanocarriers to enhance delivery specificity and facilitate cellular recognition. Wang et al. prepared the complex coacervate by CS and gelatin, which was conjugated with wheat germ agglutinin on the surface. Wheat germ agglutinin can specifically bind to the N-acetylglucosamine and sialic acid residues on GI mucosa, thus, enhancing the delivery specificity of the CS/gelatin NPs to the glycoproteins of the mucus layer of colon cancer. EGCG and 5-fluorouracil (5-FU) were co-encapsulated in these active-targeting nanocarriers, which showed enhanced cellular uptake in vitro. The EGCG/5-FU co-loaded active-targeting NPs improved the oral bioavailability of EGCG and 5-FU, as reflected by the enhanced pharmacokinetic profiles. Due to the active-targeting capacity, these NPs delivered a significantly higher amount of EGCG and 5-FU to the colon tumor. Consequently, these NPs displayed the strongest anti-colon tumor effect in vivo with less systemic toxicity compared to other types of NPs [168].

##### Topical and Transdermal Delivery

Topical delivery is also non-invasive. It provides sustained drug release and avoids the first-pass metabolism. In a phase I clinical trial, EGCG solution was topically applied to the radiation field of breast cancer patients who received radiotherapy to investigate the safety, tolerability, and preliminary effectiveness of topical EGCG for radiation dermatitis. The radiation dermatitis caused by radiotherapy was alleviated in one-third of patients after EGCG treatment [169]. Results from this phase I clinical trial indicate that topical application of catechins could be a feasible way to treat certain types of diseases.

However, the physiological structures of human skin provide barriers for topical and transdermal drug delivery. The outermost layer of human skin, the stratum corneum, is about 10–20 μm thick. The stratum corneum is mainly composed of corneocytes, which are essentially dead cells, providing a barrier for active cellular transport. The corneocytes are self-renewed constantly. Thus, they remove the unabsorbed molecules from the skin. Also, the lipid bilayers in the stratum corneum hinder the penetration of many types of molecules. Another barrier of topical and transdermal delivery is the epidermis layer, which is beneath the stratum corneum. The epidermis contains no blood vessels, thus, impedes the diffusion, permeation, and absorption of drugs [170]. These features are unfavorable for the topical and transdermal delivery of catechins.

To improve the therapeutic effects, delivery systems are introduced into the topically/transdermally applied drugs, often in combination with permeation enhancers, such as alcohols, fatty acids, and surfactants. El-Kayal et al. utilized a series of ultradeformable vesicular systems including penetration enhancer-containing vesicles (PEVs), ethosomes, and transethosomes (TEs) to encapsulate EGCG for treatment of skin cancer via topical delivery. These encapsulation systems maintained the antioxidant activity of EGCG, and at the same time, significantly enhanced the photostability of EGCG. The ethanol-containing formulations (i.e., ethosomes and TEs) displayed significantly higher intradermal deposition of EGCG than the non-ethanol-containing formulation (i.e., PEVs), with the epidermis having the highest content of EGCG followed by stratum corneum and dermis. The encapsulation systems significantly enhanced the cytotoxicity of EGCG against the A431 human epidermoid carcinoma cell line, particularly at low concentrations. Importantly, the EGCG-loaded PEVs recovered the bodyweight loss induced by the carcinogen 7,12-Dimethylbenz [α]anthouracene (DMBA). The EGCG-loaded PEVs showed excellent chemopreventative and chemotherapeutic effects against DMBA-induced skin cancer in vivo, achieving 99.61% and 98.76% tumor inhibition rate, respectively, at the end of this research, significantly higher than the pure EGCG solution. The histopathological studies revealed that EGCG-loaded PEVs maintained or restored the cellular architectures of the epidermis [171].

Liao et al. simultaneously encapsulated EGCG and docetaxel, a chemotherapeutic drug, in the nanoethosomes composed of phosphatidylcholine, ethanol, Tween-80, and sugar esters. These EGCG-containing nanoethosomes penetrated deeper than the basic nanoethosomes, primarily deposited in the hypodermis layer (60–150 μm), showing a superior transdermal permeability than the basic nanoethosomes. More importantly, the EGCG/docetaxel-containing nanoethosomes significantly inhibited melanoma growth in vivo when being topically administered [142].

EGCG was also encapsulated in the electrospun fibers [172,173,174]. These electrospun fibers have great potential as topical delivery systems for EGCG for cancer therapy, especially skin cancers.

#### 5.3.2. Invasive Delivery Strategies

The non-invasive delivery strategies are attractive and they do enhance the anticancer effects of catechins. However, the harsh GI environment, the compromised intestinal absorption, the organ metabolism, the complicated structures of human skin somewhat still limit the degree of the enhancement. It was reported that the EGCG-adsorbed nanogold delivered via intraperitoneal or intratumoral routes had better tumor growth-inhibition effects than the orally administered counterpart [175]. Thus, although non-invasive delivery strategies have high patient compliance, for the sake of better therapeutic effects, invasive delivery strategies are applied to catechins.

##### Intravenous Delivery

Intravenous (i.v.) delivery is clinically relevant. It avoids small intestine absorption and bypasses hepatic metabolism, hence, providing the highest bioavailability.

**Figure 5:**
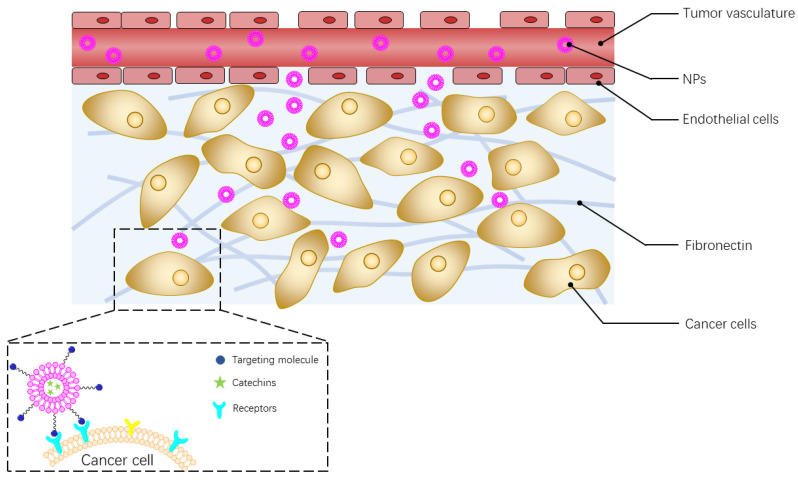
Intravenous delivery of catechin-loaded nanoparticles.

A typical application of the catechin-loaded NPs through i.v. delivery is cancer therapy. After entering systematic circulation, the catechin-loaded NPs extravasate from the blood vessel into tumor tissues due to the enhanced permeability and retention effect, which is a pathological phenomenon as tumor neovascular endothelial cells are irregularly distributed. The disorganized endothelial cells create large pores, usually between 100 and 800 nm [176], which allow NPs to extravasate into tumor tissues (Figure 5). NPs then penetrate into tumor tissues and internalize into cancer cells [177]. The payload (catechins) is preferentially released in cancer cells. It also can be released in other sites of tumor tissue (e.g., tumor stroma), particularly when stimuli-responsive nanocarriers are used [178].

Nanocarriers are often modified with targeting molecules, such as monoclonal antibodies, peptides, and aptamers [179], which can be recognized by specific receptors overexpressed on cancer cells or other cells in tumor tissue, e.g., tumor vascular endothelial cells, tumor stromal cells, and tumor-infiltrated immune cells (Figure 5). Therefore, after modification, these nanocarriers can actively target cancer cells or other cells in the tumor to achieve precise delivery of catechins. Also, it was reported that the active-targeting nanocarriers can be internalized into cancer cells through receptor-mediated endocytosis, which effectively avoids efflux of the payload from cancer cells [180], and therefore, improve the accumulation of catechins in cancer cells.

Chu et al. developed NPs composed of hyaluronic acid, fucoidan, and poly(ethylene glycol)-gelatin to co-encapsulate EGCG and curcumin [135]. These NPs can achieve dual-targeting by hyaluronic acid and fucoidan, which are the ligands of CD44 on prostate cancer cells and P-selectin on tumor vasculatures, respectively. EGCG and curcumin showed synergistic effects against PC3 human prostate cancer cells, and the EGCG/curcumin co-loaded NPs showed significantly higher cytotoxicity than pure compounds against PC3 cells. Also, the PC3 cells internalized EGCG and curcumin more efficiently from NPs than from EGCG/curcumin solution because of active targeting. Importantly, via i.v. delivery, the EGCG/curcumin co-loaded NPs displayed a potent therapeutic effect for prostate cancer in vivo and a good tumor targeting ability [135].

Other types of polymeric nanocarriers have also been used to encapsulate and deliver catechins through the i.v. route for cancer treatment (Table 4). Kazi et al. developed the folate peptide-modified PLGA NPs, which specifically target the folate receptor overexpressed in breast cancer cells, to encapsulate and actively deliver EGCG to breast cancer cells. These EGCG-loaded NPs displayed strong cellular uptake and cytotoxicity in folate receptor-expressing breast cancer cells in vitro and effectively inhibited breast tumor growth in vivo [148]. Shan et al. developed the NPs composed of EGCG, doxorubicin, and PEG-polyphenols. These i.v. delivered NPs significantly inhibited glioma growth in vivo [150].

##### Other Delivery Routes

Intratumoral (i.t.) and intraperitoneal (i.p.) administration provide loco-regional delivery of therapeutic agents, thus, enhance local drug concentration and reduce systemic side effects. Intraperitoneal delivery is advantageous for the treatment of tumors that are developed in abdominopelvic organs (e.g., liver, ovary, pancreas, colorectum, and stomach), which are coved by the peritoneum. Also, i.p. delivery is performed for the treatment of peritoneal metastasis [181,182,183]. The application of i.t. delivery is restricted by the site of the tumor, which should be reachable by injection. Also, the position of the tumor should be clear. Therefore, i.t. delivery is not as commonly used as other delivery strategies.

Hsieh et al. physically adsorbed EGCG onto the surface of nanogold particles. They found these EGCG-carrying gold NPs induced apoptosis of MBT-2 bladder cancer cells in vitro through regulation of cell cycle and expression of Bcl-2 family proteins, disruption of mitochondrial membrane integrity, and upregulation of caspase-3 and -7. Moreover, the EGCG-carrying gold NPs delivered through the i.p. or i.t. route demonstrated significant inhibition of MBT-2 bladder tumor xenograft growth through downregulation of VEGF. Interestingly, they found that the orally delivered EGCG-carrying gold NPs, although having a less potent anticancer activity than the i.p. or i.t. delivered ones, achieved the anticancer effect through relief of the immune-suppressive environment, which is different from the mechanism of the i.p. or i.t. delivered counterpart [175].

## 6. Conclusions and Future Perspective

Consumption of green tea is generally beneficial for health. As the most representative and most dominant phenolic compounds in green tea, catechins account for the majority of the health benefits. Although the anticancer activity of catechins has been proved in various in vitro and in vivo cancer models with different underlying molecular mechanisms, the anticancer activity of catechins in the human body could be limited by their low oral bioavailability. The administration of catechins encapsulated in nanodelivery systems through different routes has shown encouraging outcomes in enhancing the anticancer effect in in vivo models. Catechins are natural phenolic compounds that have relatively lower systemic toxicity than chemotherapeutic drugs, these catechin-loaded NPs are promising agents with low side effects for cancer therapy.

As mentioned in the previous section, gut microbiota plays an important role in cancer development. Catechins that are not absorbed from the small intestine go to the large intestine where they interact with the microbes to perform the anticancer activity. Therefore, the design of nanodelivery systems should not only focus on enhancing the small intestinal permeability of catechins. Efforts could also be devoted to enhancing the amount of catechins that can be delivered to the microbiota, and a step further, be precisely delivered to the specific microbes to achieve the anticancer effect. Research in this area is barely reported so far. Hence, it is one of the directions for future research of catechin nanodelivery.

Using nanodelivery strategies to enhance the anticancer activity of catechins is promising in preclinical studies. However, the nanoformulated catechins have not entered clinical trials for evaluation so far. Hence, it should be realized that there is still a huge amount of work that should be done before these nanoformulated catechins can be used in cancer patients for treatment. Efforts should be continuously made to facilitate breakthroughs in this area.

## Figures and Tables

**Table 1:** Mechanisms of cancer inhibitory effects of green tea catechins.

Type of Cancer	Catechin	Experimental Models	Inhibition Mechanisms	Ref.
Lung Cancer	EGCG	A549 cell	Inhibit activation of p300/CBP in TGF-β1-induced EMT by deacetylation of Smad2 and Smad3	[58]
	EGCG	A549 and H1299 cells	Inhibit TGF-β-induced EMT through downregulation of phosphorylated Smad2 and Erk1/2	[59]
	EGCG	CL1-5 cell and CL1-5 tumor-bearing nude mice	Downregulate the expression of MMP-2 via the JNK pathway, induce G2/M arrest	[81]
	EGCG	Tumorspheres derived from A549 and H1299 cells	Inhibit CSC through suppression of the Wnt/β-catenin pathway	[66]
	C, EC, ECG, EGC, EGCG, and mixture	A549 cell	EGCG induces apoptosis through a p53-dependent pathway	[82]
Breast Cancer	EGCG	MDA-MB-231 cell	Block the Wnt pathway by inducing the HBP1 transcriptional repressor	[83]
	EGCG	MCF-7 cell	Inhibit the gelatinolytic activity and expression of MMP-2 by downregulating MT1-MMP, VEGF, NF-κB, FAK, αvβ3, and α5β1 integrin receptors	[84]
	EGCG	MDA-MB-231 cell	Inhibit proliferation by inactivation of the PI3K/AKT/mTOR pathway	[85]
	EGCG	MCF-7 cell	Inhibit proliferation and promote apoptosis via p53/Bcl-2 pathway	[86]
	EGCG	MDA-MB-231 cell	Inhibit cell growth via inactivation of β-catenin pathway	[87]
Ovarian Cancer	EGCG	SKOV-3, OVCAR-3, PA-1 cells	Induce apoptosis and arrest cell cycle	[88]
	EGCG	SKOV3 cell	Inhibit proliferation and induce apoptosis, downregulate of aquaporin 5 via inactivation of NF-κB	[89]
	EGCG	SKOV-3 cell	Inhibit proliferation via DNA synthesis reduction, induce apoptosis via DNA damage, arrest cell cycle	[90]
Gastric Cancer	EGCG	SGC-7901 cell and SGC-7901 tumor-bearing nude mice	Inhibit angiogenesis and the expression of VEGF, reduce the activation of Stat3	[91]
	EGCG	AGS and SGC7901 cells	Inhibit proliferation by regulating the long non-coding RNA LINC00511/miR-29b/KDM2A axis	[92]
	EGCG	SGC7901 cell	Induce apoptosis under hypoxia via downregulation of HIF-1α and VEGF	[93]
Colon Cancer	EGCG	HT-29 cell	Inhibit COX-2 through activation of the AMPK pathway	[40]
	EGCG	HT-29 cell	Induce apoptosis through Akt, ERK1/2, and alternative p38MAPK pathways	[94]
	EGCG	HT-29 cell	Induce cell cycle arrest, apoptosis, and autophagy	[95]
	EGCG	Caco2, HCT116, HT29, SW480, SW837 cells	Inactivation of the insulin-like growth factor-1 receptor	[96]
Pancreatic Cancer	EGCG	Colo357 cell	Inhibit IL-1-induced secretion of IL-6, IL-8, VEGF, and PGHS-2, reduce the level of MMP-2, activate caspase-3, downregulate the expression of IL-1 receptor type I via inhibition of NF-κB	[97]
	CG, ECG, EGCG	PancTu-I, Panc1, Panc89, BxPC3 cells	Arrest cell cycle, inhibit TNF-α-mediated activation of NF-κB and secretion of IL-8 and uPA	[98]
	EGCG	Panc-1, MIA PaCa-2, BxPC-3, HPAF-II, CFPAC-1, Su.86.86, FC1245 cells, and FC1245 tumor-bearing mice	Inhibit migration and invasion by suppressing EMT via inhibition of the Akt pathway	[60]
Liver Cancer	EGCG	HLE, HepG2, HuH-7, PLC/PRF/5 cells, HLE tumor-bearing nude mice	Induce apoptosis, downregulate Bcl-2α and Bcl-xl via inactivation of NF-κB	[99]
	EGCG	HLF, PLC/PRF/5, HepG2, HLE, Hep3B, HuH-7 cells, and HuH-7 tumor-bearing nude mice	Inhibit the VEGF-VEGFR axis and downstream signaling molecules (ERK, Akt), downregulate the expression of Bcl-xL	[100]
	EGCG	HepG2 and Huh7 cell, diethylnitrosamine-induced liver cancer rat model	Inhibit proliferation, downregulate the expression of cell division cycle 25A, upregulate the expression of p21waf1/Cip1	[101]
Bladder Cancer	EGCG	SW780 cell and SW780 tumor-bearing nude mice	Inhibit proliferation and migration via downregulation of NF-κB and MMP-9	[102]
	EGCG	Tumorspheres derived from EJ and UM-UC-3 cells	Inhibit CSC through suppression of the Hedgehog pathway	[68]
Prostate Cancer	EGCG	PC-3 cell	Inhibit proliferation by activation of ERK1/2 via a MEK-independent, PI3K-dependent pathway	[103]
	EGCG	LNCaP cell sublines and LNCaP104-R1 tumor-bearing nude mice	Suppress cell proliferation, prostate-specific antigen expression, and androgen receptor transcriptional activity	[104]

VEGFR: vascular endothelial growth factor receptor.

**Table 2:** Parameters related to intestinal permeability of major catechins.

Catechins	M.W. (g/mol)	Log P ^a^	H-Bond Donor	H-Bond Acceptor	P_app_ × 10^−7^ (cm/s) ^b^		
					AP to BL	BL to AP	Efflux Ratio
EC	290	1.5	5	6	1.39	29.96	21.55
EGC	306	1.11	6	7	1.49	7.72	5.18
ECG	442	2.46	7	10	0.96	3.86	4.02
EGCG	458	2.07	8	11	0.83	1.52	1.83

^a^ Calculated by ChemDraw 19.0. ^b^ Assessed by Caco-2 monolayer transport experiment. Efflux ratio = P_app_ (BL to AP)/P_app_ (AP to BL) [107]. AP: apical; BL: basolateral; M.W.: molecular weight; P_app_: apparent permeability coefficient.

**Table 3:** Improvements of catechins encapsulated in nanocarriers.

Nanocarriers	Catechins	Improvements	Ref.
β-lactoglobulin NPs	EGCG	Preserved the antioxidant activity of EGCG at neutral pH	[119]
β-lactoglobulin NPs	EGCG	Enhanced anticancer activity of EGCG in vitro	[118]
Gelatin NPs	Tea polyphenols	Enhanced sustained release of tea polyphenols in vitro, significantly improved the pharmacokinetic profiles and oral bioavailability of tea polyphenols in vivo	[120]
Hordein NPs	EGCG	Protected EGCG from degradation	[121]
CS/TPP complex NPs	C and EGCG	Enhanced stability of C and EGCG in pH 7.4 buffer	[122]
CS/TPP complex NPs	EGCG	Significantly inhibited prostate tumor growth in vivo	[123]
CS/TPP complex NPs	EGCG	Enhanced the anti-melanoma effect of EGCG in vitro and in vivo	[124]
CS/TPP complex NPs modified with PEG and folate	EGCG	Enhanced anticancer effect of EGCG against MCF-7 cells by regulating the PI3K/Akt pathway	[125]
CS/β-lactoglobulin complex NPs	EGCG	Enhanced cellular antioxidant activity of EGCG	[126]
CS/gelatin complex NPs	EGCG	Significantly decreased the expression of VEGF in gastric cancer cells and significantly inhibited gastric tumor growth in vivo	[127]
β-lactoglobulin/gum Arabic complex NPs	EGCG	Enhanced antioxidant activity of EGCG	[128]
Ovalbumin-dextran conjugate NPs	EGCG	Significantly enhanced the P_app_ of EGCG in vitro	[129]
CS-coated BSA NPs	Tea polyphenols	Significantly enhanced the radioprotective effect in vivo	[130]
Poly-ε-lysine- or CS-coated BSA NPs	EGCG	Significantly enhanced the Papp of EGCG in the CS-coated EGCG-loaded BSA NPs compared to the EGCG solution	[131]
Folate conjugated CS NPs	EGCG	Effectively enhanced anticancer effect in 3 cancer cell lines, especially in the folate receptor-overexpressing cell line	[132]
CS/PAA NPs	EGCG	Increased the stability of EGCG in simulated gastric and intestinal conditions, significantly enhanced the oral anti-atherosclerosis effect in rabbit in vivo through the reduction of serum levels of triglyceride, total cholesterol, HDL cholesterol, and LDL cholesterol	[133]
CS/γ-PGA	Tea catechins	Effectively enhanced the in vitro transport of tea catechins through Caco-2 monolayer	[134]
Hyaluronic acid/fucoidan/PEG-gelatin NPs	EGCG	Significantly enhanced the inhibitory effect against prostate cancer in vitro and in vivo	[135]
SLN	EGCG	Significantly enhanced in vitro cytotoxicity against human breast cancer cells MDA-MB-231 and human prostate cancer cells DU-145 through apoptosis	[136]
SLN	EGCG	Enhanced stability of EGCG	[137]
Nanoliposome	Tea polyphenol	Enhanced tea polyphenol stability in pH 7.4 buffer	[138]
CS-coated nanoliposome	EGCG	Significantly enhanced EGCG stability, improved sustained-release, increased intracellular level of EGCG in MCF-7 cells, induced apoptosis, and inhibited proliferation of MCF-7 cells	[139]
Nanostructured lipid carriers and CS-coated nanostructured lipid carriers	EGCG	Significantly enhanced the stability of EGCG at pH 7.4, increased the sustained release of EGCG and the content of EGCG in the macrophage, enhanced the anti-atherogenic activity of EGCG by the reduction of the cholesteryl ester content in the macrophage, and significant inhibition of inflammatory factor secretion	[140]
RGD peptide-modified nanostructured lipid carriers	EGCG	Enhanced in vitro cytotoxicity of EGCG against breast cancer cells	[141]
Nanoethosomes	EGCG	Significantly enhanced melanoma growth-inhibition in vivo when being topically delivered	[142]

BSA: bovine serum albumin; HDL: high-density lipoprotein; LDL: low-density lipoprotein; PAA: polyaspartic acid; γ-PGA: poly(γ-glutamic acid); TPP: tripolyphosphate.

**Table 4:** Enhancing anticancer effect of catechins with invasive nanodelivery strategies.

Delivery Strategy	Nanocarrier	Targeting Molecule	Receptor	Catechin	Co-Delivered Drug	Cancer Type	Models	Efficacies	Ref.
i.v.	PLGA NPs	Folate peptide	Folate receptor	EGCG	-	Breast cancer	MDA-MB-231 and MCF-7 cell lines, SD rats and MDA-MB-231 tumor-bearing nude mice	↑ In vitro cellular uptake of NPs in the folate receptor-expressing breast cancer cell lines ↑ In vitro cytotoxicity against the folate receptor-expressing breast cancer cell lines compared to free EGCG and non-targeting counterpart ↑ Percentage of early and late apoptotic cells in vitro, which was related to an elevated level of ROS, loss of mitochondrial membrane potential, downregulation of Bcl-2, and upregulation of Bax ↑ In vivo pharmacokinetic profiles and tumor accumulation ↑ Inhibition of breast tumor growth in vivo	[148]
i.v.	TPGS-g-HA/FD/PEG-g-gelatin NPs	HA and FD	CD44 and L- or P-selectins	EGCG	Docetaxel	Prostate cancer	PC3 cell line and PC3 tumor-bearing mice	↑ Anticancer effect in vitro ↑ Cell cycle arrest ↑ Inhibition of tumor growth in vivo	[149]
i.v.	Fe^3+^-doxorubicin@EGCG-PEG NPs	-	-	EGCG	Doxorubicin	Glioma	U87MG and 293T cell lines, Balb/C mice, and U87MG tumor-bearing mice	↓ Expression of carbonyl reductase 1 and generation of doxorubicinol ↑ in vitro cytotoxicity against cancer cells, and penetration into multicellular spheroids ↑ Tumor accumulation and blood circulation ↓ Hematotoxicity and cardiac toxicity in vivo ↑ Inhibition of tumor growth in vivo	[150]
i.t.	PEI/pDNA/HA-EGCG ternary complexes	HA	CD44	EGCG	pDNA	Colon cancer	HCT-116 and HEK293 cell lines and HCT-116 tumor-bearing mice	↑ Gene transfection efficiency in vitro ↑ Cellular uptake in CD44-overexpressing cells ↑ Tumor accumulation in vivo	[151]
i.p.	PLGA NPs	-	-	EGCG	-	Lung cancer	A549 and H1299 cell lines and patient-derived xenograft bearing nude mice	↑ In vitro cellular uptake in lung cancer cells ↑ In vitro cytotoxicity against lung cancer cells ↑ Lung cancer cell apoptosis by suppression of NF-κB ↑ Inhibition of lung cancer PDX growth in vivo by enhancing apoptosis, inhibiting proliferation, and downregulation of phosphor-NF-κB	[147]
i.p.	Folate and PEG mod ied CS/TPP complex NPs	Folate	Folate receptor	EGCG	-	Breast cancer	MCF-7, MDA-MB-231, HCC-70, 4T1, Panc-1 cell lines, MDA-MB-231 mammosphere, and MDA-MB-231 tumor-bearing nude mice	↑ In vitro cellular uptake ↑ Expression of CCN5 in vitro ↑ Tumor growth in vivo	[152]

FD: fucoidan; HA: hyaluronic acid; PDX: patient-derived xenograft; pDNA: plasmid DNA; PEG: polyethylene glycol; PEI: polyethylenimine; TPGS: D-α-tocopheryl polyethylene glycol 1000 succinate.

## Data Availability

The data presented in this study are available on request from the corresponding author.

## Abbreviations

	**Full Name**
5-FU	5-Fluorouracil
AP	Apical
AP-1	Activator protein-1
ARE	Antioxidant response element
AUC	Area under the curve
BSA	Bovine serum albumin
BL	Basolateral
C	(+)-catechin
CDKs	Cyclins-dependent kinases
CG	Catechin gallate
C_max_	Maximum plasma concentration
COMT	Catechol-*O*-methyltransferase
CYP450	Cytochrome P450
CYP1A1	CYP450 1A1
CS	Chitosan
CSCs	Cancer stem cells
DMBA	7,12-Dimethylbenz [α]anthouracene
EC	(−)-Epicatechin
ECDG	Epicatechin digallates
ECG	(−)-Epicatechin gallate
EGC	(−)-Epigallocatechin
EGCDG	Epigallocatechin digallates
EGCG	(−)-Epigallocatechin gallate
EMT	Epithelial-mesenchymal transition
FD	Fucoidan
GC	Gallocatechin
GCG	Gallocatechin gallate
GI	Gastrointestinal
HA	Hyaluronic acid
HDL	High-density lipoprotein
IL	Interleukin
i.p.	Intraperitoneal
i.t.	Intratumoral
i.v.	Intravenous
Keap1	Kelch-like ECH-associated protein 1
LDL	Low-density lipoprotein
LOX	Lipoxygenases
MMP	Matrix metalloproteinase
M.W.	Molecular weight
NF-E2	Nuclear factor-erythroid 2p45
NF-κB	Nuclear factor-κB
NPs	Nanoparticles
Nrf2	NF-E2-related factor 2
PAA	Polyaspartic acid
P_app_	Apparent permeability coefficients
PARP	Poly (ADP-ribose) polymerase
PCNA	Proliferating cell nuclear antigen
pDNA	Plasmid DNA
PDX	Patient-derived xenograft
PEG	Polyethylene glycol
PEI	Polyethylenimine
PEVs	Penetration enhancer-containing vesicles
γ-PGA	Poly(γ-glutamic acid)
PI3K	Phosphoinositide-3-kinase
PLGA	Poly(lactic-co-glycolic acid)
RNS	Reactive nitrogen species
ROS	Reactive oxygen species
SLNs	Solid lipid nanoparticles
TEs	Transethosomes
TNF	Tumor necrosis factor
TPGS	D-α-tocopheryl polyethylene glycol 1000 succinate
TPP	Tripolyphosphate
VEGF	Vascular endothelial growth factor
VEGFR	Vascular endothelial growth factor receptor
